# The General and Special Medical Societies of America

**Published:** 1891-11

**Authors:** Adam H. Wright

**Affiliations:** Toronto


Buffalo Medical and Surgical Journal

Vol. XXXI. NOVEMBER, 1891. No. 4.

### Original Communications.

### THE GENERAL AND SPECIAL MEDICAL SOCIETIES OF AMERICA.[1]

By ADAM H. WRIGHT, M. D., Toronto.

When we separated after our pleasant and profitable meeting in Philadelphia last September, it was expected that we would meet this year in Washington as members of the Congress of American Physicians and Surgeons. The Congress, however, has refused to admit us, and, therefore, we are not going to Washington.

I desire, with the permission of our executive council, to place on record a plain statement of facts respecting the negotiations which have been carried on between that body and our Association. In the year 1886, after careful consideration on the part of certain representative physicians and surgeons of the United States, it was decided to form such a Congress. Preliminary invitations were sent to the various special societies, asking for their assistance and coöperation in the new undertaking. All the societies then in existence, except one, returned favorable replies. The American Gynecological Society alone refused to coöperate. In the report of the meeting of this society for 1887, which appeared in the *New York Medical Record*, we find the following words: "The proposition to become a part of the American Congress of Physicians and Surgeons was not adopted." The promoters of the proposed confederation were naturally disappointed and considerably discouraged by the action of this strong and able society. They desired a representation of the important subjects of obstetrics and gynecology. After a conference, some strong friends of the Congress decided to organize a new society of obstetricians

1. The President's annual address to the American Association of Obstetricians and Gynecologists, delivered at the fourth annual meeting held at the New York Academy of Medicine, September. 1891.

and gynecologists. A preliminary meeting of a number of prominent obstetricians and gynecologists was held in Buffalo, April 19, 1888, with that object in view, and the result was that the American Association of Obstetricians and Gynecologists was organized, not in opposition to any other society in any sense, but largely in the interest of the new Congress. For some months these promoters of our Association did some prodigious work in their efforts to perfect the organization. I have been advised to mention no names in this connection, and I rather regret that I have acted on such advice, because I feel that I am scarcely doing justice to men who worked quietly but persistently for many weary months to make the Association a credit alike to the continent of America and the Congress which it expected to enter.

In due time our organization was fairly completed. Those who had worked so faithfully and so unselfishly began to feel that their efforts had been crowned with success. A formal application for admission was sent to the Congress. In the meantime, however, a change had come over the society, which had formerly opposed the proposed Congress. Whether this marvelous change was brought about by our organization, I know not; but it was a singular coincidence that the applications from the old Gynecological Society and the new Association of Obstetricians and Gynecologists for admission to the Congress, were practically made at one and the same time. After some deliberation by the executive authorities of the Congress, it was decided that the society which had shown pronounced hostility to the Congress up to the date of its sudden conversion and application for admission should be received, and that the new organization, which had been formed to assist the confederation in a serious emergency, should be put on trial for a couple of years. In accordance with this remarkable decision, the following resolution was passed:

"*Resolved*, That it is the sense of this Executive Committee that they will not consider the application of any society which has not held at least two annual meetings."

This decision was received with a certain amount of surprise, but with becoming meekness and humility; and we entered into our period of probation with some feelings of disappointment, but with strong hopes that our work would be judged on its merits, and duly recognized at the proper time. At the end of our second year we felt extremely gratified at the work which had been accomplished by our members. We felt certain that our two published volumes of Transactions would quite fulfil the requirements which had been exacted from us. These volumes were duly filed, substantially constituting our second application, to which we received no reply for many months.

Shortly after our meeting in Philadelphia we received a communication asking for twelve copies of each volume of our Transactions. This was a third surprise and disappointment to us, but we presumed that the request was made in good faith, and we acted accordingly. Our third meeting was so successful from every point of view, that we thought a perusal of the Transactions would strengthen any favorable impressions which had been created by the former two. It was, to a certain extent, embarrassing to us, because we were unable to announce definitely the time and place of our next meeting. Our third volume was completed as soon as possible, and the thirty-six books forwarded to the Executive Committee of the Congress. When all the evidence as to our position was received, the committee did not arrive at a conclusion suddenly or rashly; they took ample time for deliberation, and while they were deliberating we were waiting. Month after month dragged along, and still the decision came not. The patience of our Council during these months reached a sublimity which appeared to me almost ridiculous. At last, a meeting of the Executive Committee of the Congress of American Physicians and Surgeons was held in Philadelphia, April 26, 1891, and shortly we received an unofficial intimation that we would not be admitted to the Congress.

I have endeavored to give you a plain statement of what appears to me one of the most extraordinary transactions known to medical history. The question naturally arises, Why were we accorded such treatment? I am unable to answer. A rumor has reached me to the effect that the chief argument used against us was that our Association really represented nothing more than a duplication of the work of other sections, and for that reason should not be admitted. I have nothing to do with such an argument, and care not whether it be considered good, bad, or indifferent. I will remove the necessity of using it by saying that we concede that the Congress had a perfect right to refuse to admit us if its members thought fit. We insist, however, that it had no right to subject us to humiliation such as this; it had no right to place us on probation for an extended period, and then absolutely ignore the essence of the implied contract. The resolution of the Congress required certain things from us. We have fulfilled those requirements in every particular. We actually came into existence in the interest of the Congress; we have supported it loyally in every particular; we have shown no particle of antagonism to any of its sections; we have, as a matter of fact, patiently submitted to much inconvenience through the delay in sending its singular ultimatum. Is it possible that the majority of the members of that great organization will feel proud of the actions of their executive? I have considered the matter in all its aspects, and I cannot conceive how the members of the Congress can reasonably defend the methods of their committee.

Well, gentlemen, what are we now to do? It gives me unbounded pleasure to assure you that our Executive Council holds no divided opinions. The necessities of the case compel us to bid the Congress a sad farewell, but in doing so we indulge in the hope that we may be permitted to continue our existence, which we have found exceedingly pleasant as well as extremely profitable. Our Association is alive to-day; it is going to live; it is going to thrive; it is going to do a great work on this vast continent. I say this in no boasting spirit. I desire to assume no air of bravado. I feel fully impressed with the responsibility I assume when I say that we have a grand future before us. I, who have done so little for you, can express myself with greater freedom than could others who have borne so nobly the burden of organizing this magnificent society. I have witnessed the efforts of our founders with profound admiration; I have watched their zeal, their devotion, their untiring energy, with a feeling of wonder; I have viewed their boundless enthusiasm, their wondrous capacities for work, and their unselfish devotion to each other and our common cause, with perfect delight. In addition, it gives me great pleasure to refer to the dignified bearing of our councilors under somewhat trying circumstances. In our negotiations with the Congress, I know of no act on our part that will ever bring the blush of shame to any of our members. While referring to the actions of our office-bearers, I cannot refrain from also referring to the loyal support they have ever received from the ordinary Fellows of our Association. It appears to me that our prospects were never brighter than they are today. The main object of our Association, "the cultivation and promotion of knowledge in whatever relates to abdominal surgery, obstetrics and gynecology," is ever kept in view by one and all; and the results in three short years, the evidence of which may be found in three volumes of our Transactions, are such as will inspire us with confidence and fill us with hope in the future.

Let it be our duty, as well as our pleasure, to worthily continue the work which has been so auspiciously begun. Let envy, hatred, and all uncharitableness toward other societies be ever kept far from us. Let us forget the indignities that have been heaped upon us. Let our memories of the past pertaining to our own work ever remain as pleasant as they are today.

It gives me pleasure and satisfaction to call attention to the fact that, geographically speaking, this Association is American in the broadest sense of the word. As our President of last year expressed it, "the Association is not limited to the United States, but only by the boundaries of the Western Continent." I know of no other medical society in existence that is essentially continental in character, and I am glad to be able to assure you that this feature of the organization is highly appreciated by my countrymen in the goodly-sized Dominion north of this flourishing Republic. It happens by an unfortunate coincidence that the meeting of the Canadian Medical Association is being held in Montreal concurrently with this. As we desired to treat that Society with no discourtesy, we have had but little to say about this meeting in Canada. I am much gratified, however, that Dr. Ross and myself have been able to propose the names of some Canadians, and I am pleased that you have been good enough to accept them to membership without putting them on probation for two or three years.

I would like to make a few remarks on the subject of medical societies, with a view to our position at the present juncture; and I do so with considerable diffidence, because my opinions may be distasteful to some of our Fellows, and to some warm friends outside. My remarks, however, will simply represent my own individual views, and you may take them for what they are worth.

We are now perfectly free and untrammeled in every respect, and it may be well to consider what position we should assume in regard to other societies. Probably all will admit the possibilities of great dangers arising out of specialties. This subject has caused considerable discussion in all parts of the world during recent years. It is not my purpose to discuss the general aspects of the subject now; but in reference to societies, I would regret very much to see a too-well-marked line of demarcation between the specialists and the great mass of general practitioners of this country. What should be the greatest medical organizations in the United States and Canada respectively? In my opinion, the American Medical Association and the Canadian Medical Association. I have long had a very decided opinion that the greatest medical organization that has ever existed is the British Medical Association. The great mass of British practitioners of all sorts and conditions belong to it, and take pride in their membership. It contains no less than thirteen thousand eight hundred members. At the recent meeting at Bournemouth, a large portion of the brightest lights of Great Britain were present, and devoted their best energies toward making the meeting a success. This is the rule from year to year, and the great society is growing with wondrous rapidity. In this country it seems to me that too many of the leaders of the profession are conspicuous by their absence from the meetings of the American Medical Association. Many of the men referred to are the peers of the best men that can be found in any nation or any clime. Is it not unfortunate that so many of them miss what should be the best opportunity of meeting the rank and file of the profession in their own country? Is there no remedy for this unfortunate condition of things? It would seem to me that all should unite to make the national society the greatest in the land, and that all local and special societies should cordially work together for that purpose. I am pleased to note that many of our Fellows are enthusiastic workers in the American Medical Association, and I hope that they and all others will give it at least as loyal a support in the future as they have in the past.

I was particularly struck recently with an example of the great good which can be accomplished by a meeting of specialists with general practitioners. I had the privilege of attending a very excellent meeting of the Medical Society of the State of New York in February last; and among the many good things there was an admirable discussion on the important subject of appendicitis, valuable alike to those who talked and those who listened. Operations, when required for this condition, may be relegated to those who pay special attention to abdominal surgery, but the best methods of diagnosis should be known to all. The various phases of the question were so thoroughly and ably discussed, that the large assemblage was duly impressed with the importance and correctness of the views expressed. It is impossible to put any proper estimate on the good that may be accomplished by such meetings and discussions. The benefits are not confined to one side, but extend alike to specialists and general practitioners. In speaking of specialists, I may say I refer to those who pay special attention to such subjects as obstetrics and gynecology, whether their work be entirely confined to these departments or not. For such, the benefits to be derived by personal contact with intelligent, industrious, and observing general practitioners are incalculable. Such association will do much to keep the specialists from becoming narrow, priggish, dogmatic, and—may I say it ?—dangerous.

We meet today in this great city as a society of physicians and surgeons, who may be called specialists in the sense I have indicated. Of course, modern gynecology is understood to include abdominal surgery, which has made such wondrous advances in recent years. I think our work on this continent in these different departments will compare favorably with that done in older countries. A few short years ago the results of our efforts in abdominal surgery were somewhat discouraging. Our mortality rates compared very unfavorably with those of Great Britain and the Continent. Some of the reasons given for this condition of things were positively ludicrous. The history of the evolution of this branch of surgery is so recent as to have been almost the talk of yesterday, and I need not dilate upon it today. What seem more simple now than our methods of cleanliness in surgery— and yet how hard they were to learn ! I think, however, I am justified in saying that we have conquered our former serious difficulties, and our high mortalities of from 15 to 50 per cent. in our various kinds of abdominal sections have vanished, I hope, forever.

Our advances in obstetrics, although not so brilliant as those to which I have just alluded, have been quite as valuable, and are exactly in the same line. And yet we have found it difficult to learn, and to teach, that obstetricians should be as scrupulously careful in their methods as abdominal surgeons. I may say, in addition, that this lesson has not yet been learned by a large proportion of our physicians, and we must go on preaching and teaching cleanliness to all our practitioners until it is recognized practically as a criminal offense to neglect to clean the hands, the instruments, and the surroundings of our patients in labor. We are proud of the fact that we have almost driven septicemia from properly conducted maternity hospitals. Let us drive it away from this continent altogether.

In making any allusions to the past year, I am very thankful that I have no obituary references to make respecting our Fellows. Some, however, have been sorely afflicted by family bereavement, and when our brothers suffer we feel in a sense a certain amount of the weight falling on our own shoulders. Of such matters I shall make no special mention, excepting in one instance. I desire to give expression to the profound sorrow we all feel in consequence of the almost appalling calamity which has befallen our Secretary in the loss of his only—his well-beloved—son, who was alike an ornament to our profession, a worthy citizen of this Republic, a loving son, husband and father, and a most charming and estimable man. What William Warren Potter has done for this Association cannot be told, but has, I am glad to say, been very highly appreciated by all. We know that since the inception of our organization he has given us the benefit of some of the best efforts of his life. We all respect and admire him for what he has done for us; we love him for his rare personal qualities and goodness of heart; we extend to him in his hour of tribulation our warmest sympathies, and our best wishes for him and his for all time to come.

I cannot refrain from making reference to the great loss this country has sustained in the death of Dr. Fordyce Barker. He was not simply a distinguished obstetrician of this continent—he was known abroad almost as well as he was at home—he was one whom the whole medical world admired, respected, and delighted to honor. It was my pleasure and privilege to listen to some of his lectures in my student days, and since that period to read everything from his pen that I could find; and I feel that I am indebted to him for some of the most valuable lessons I have ever learned in the science and art of obstetrics. I desire, therefore, to offer my humble tribute, in addition to the almost countless others from all parts of the globe, to the memory of one of the grandest and noblest physicians the world has ever seen.

In conclusion, I must confess that your choice of a President last year at Philadelphia was more creditable to your good nature than your better judgment. I feel constrained, however, to forgive you for that mistake, and to thank you for myself and for my country. I cannot express the satisfaction it would afford me if any words of mine would encourage you to work with still more vigor and enthusiasm in the future than you have in the past. I feel, however, that that is unnecessary. I think that our Association is not composed of the material that is likely to weaken in the hour of trial. I trust that the troubles that have beset us will be the means of forming the strongest link in the firm chain that binds us together as brothers and co-workers in a good, a great cause.

